# Increased knee laxity with hamstring tendon autograft compared to patellar tendon autograft: a cohort study of 5462 patients with primary anterior cruciate ligament reconstruction

**DOI:** 10.1007/s00167-018-5029-9

**Published:** 2018-06-28

**Authors:** Riccardo Cristiani, Vasileios Sarakatsianos, Björn Engström, Kristian Samuelsson, Magnus Forssblad, Anders Stålman

**Affiliations:** 1 Department of Molecular Medicine and Surgery, Stockholm Sports Trauma Research Center, Karolinska Institutet, Solna, Sweden; 2 Capio Artro Clinic, Sophiahemmet Hospital, Valhallavägen 91, 11486 Stockholm, Sweden; 3 Department of Orthopaedics, Institute of Clinical Sciences, The Sahlgrenska Academy, University of Gothenburg, Gothenburg, Sweden; 4 Department of Orthopaedics, Sahlgrenska University Hospital, Mölndal, Sweden

**Keywords:** Anterior cruciate ligament, ACL reconstruction, Hamstring, Patellar tendon, Graft, Laxity, Outcome, KOOS, Lysholm

## Abstract

**Purpose:**

To compare anterior knee laxity and patient-reported outcome measures (PROMs) between anterior cruciate ligament reconstruction (ACLR) performed with bone–patellar tendon–bone (BPTB) and hamstring tendon (HT) autografts and, moreover, to study any correlation between postoperative anterior knee laxity and PROMs.

**Methods:**

Patients who underwent primary ACLR at Capio Artro Clinic, Stockholm, Sweden, from January 2000 to October 2015, were identified in our local database. Instrumented laxity measurements and PROMs were reviewed. The KT-1000 arthrometer, with an anterior tibial load of 134-N, was used to evaluate knee laxity preoperatively and at the 6-month follow-up. The Lysholm score was collected preoperatively and at 6 months postoperatively. The Knee injury and Osteoarthritis Outcome Score (KOOS) was collected preoperatively and at the 1-year follow-up.

**Results:**

A total of 5462 primary ACLRs, 692 BPTBs and 4770 HT autografts were included in the study. All the patients showed a significant reduction in knee laxity from preoperatively to postoperatively (BPTB group: from 3.8 ± 2.6 to 1.2 ± 2.1 mm; HT group: from 3.6 ± 3.1 to 1.8 ± 2.2 mm; *P* < 0.001 for both). The HT group showed a significantly increased postoperative knee laxity compared with the BPTB group (1.8 ± 2.2 vs 1.2 ± 2.1 mm; *P* < 0.001). The mean anterior tibial translation (ATT) reduction from preoperative to postoperative was significantly larger for the BPTB graft compared with the HT graft (2.7 ± 2.2 vs 1.7 ± 2.6 mm; *P* < 0.001). A significantly higher rate of “surgical failures”, defined as a postoperative side-to-side (STS) difference > 5 mm, was found in the HT group compared with the BPTB group at follow-up (4.3 vs 2.4%; *P* < 0.001). A significantly larger improvement was found in the HT group compared with the BPTB group for the KOOS Pain (9.5 vs 8.0; *P* = 0.02), Activities of Daily Living (7.2 vs 5.7; *P* = 0.006), Sports (24.2 vs 15.3; *P* < 0.001) and Quality of Life (25.8 vs 22.1; *P* = 0.001) subscales. No significant difference regarding the mean improvement in the Lysholm knee score was found between the two grafts (BPTB group: 14.5, HT group: 14.0; n.s.). No correlation between postoperative anterior knee laxity and PROMs was found in either graft group.

**Conclusion:**

Primary ACLR performed with HT autograft resulted in greater postoperative anterior knee laxity and significantly more surgical failures (STS > 5 mm) compared with BPTB autograft. The BPTB autograft showed a larger anterior knee laxity reduction (ATT reduction) in conjunction with primary ACLR. The HT autograft led to a significantly larger improvement in four of five KOOS subscales from preoperatively to the 1-year follow-up, compared with BPTB autograft. There was no association between postoperative anterior knee laxity and PROMs for either graft. The findings of the present study provide clinicians with valuable information regarding differences in knee laxity and subjective knee function between BPTB and HT autograft after primary ACLR. The use of BPTB autograft should be considered for patients with high knee stability demands.

**Level of evidence:**

Retrospective cohort study, Level III.

## Introduction

An anterior cruciate ligament (ACL) tear often leads to functional knee instability, interfering with sports and activities of daily living [4, 16]. An ACL-deficient knee predisposes to meniscal and cartilage tears, increasing the risk of the early onset of osteoarthritis [7, 36]. Restoring knee joint laxity, to improve subjective knee function, is the primary goal of ACL reconstruction (ACLR). The most commonly used autografts for ACLR are bone–patellar tendon–bone (BPTB) and hamstring tendons (HT) [10, 28]. Numerous studies have compared the two grafts for ACLR, but the choice of graft still remains controversial. During the last decade, the HT autograft has been used more frequently for primary ACLR [32]. The BPTB autograft has been associated with more donor-site morbidity problems, such as anterior knee pain, kneeling pain and extension loss, compared with the HT autograft [2, 18, 29, 45]. On the other hand, ACLR performed using HT autograft showed significant flexor torque deficiency compared with BPTB autograft [5, 23]. There is controversy in the literature regarding the potential of these grafts to restore knee laxity. Some authors have found increased anterior knee laxity using HT autograft [3, 5, 9, 12, 13]. Conversely, in other studies, no differences in terms of postoperative anterior knee laxity were found between BPTB and HT autograft [15, 25, 28, 43].

The primary aim of the present work was to evaluate any differences in anterior knee laxity in ACLRs performed with BPTB or HT autograft. The second aim was to compare patient-reported outcome measures (PROMs) between the two autografts. The third aim was to investigate any correlation between postoperative anterior knee laxity and PROMs. It was hypothesized that (a) postoperative anterior knee laxity is greater after ACLR performed with HT graft compared with BPTB graft; (b) there are no significant differences in terms of PROMs between the two grafts; (c) greater postoperative anterior knee laxity results in poorer PROMs.

## Materials and methods

Patient data were extracted from our local database at the Capio Artro Clinic, Stockholm, Sweden. Inclusion criteria were set to patients who underwent primary single-bundle ACLR with no concomitant ligament injuries, using either HT or BPTB autograft, in the 15-year period ranging from January 2000 to October 2015 (*n* = 7185). The exclusion criteria were contralateral ACL injuries or reconstruction (*n* = 274) and no pre- or postoperative KT-1000 arthrometer (MEDmetric, Corp., San Diego, CA, USA) values available (*n* = 1449).

### Surgical technique and rehabilitation

For all patients, a single-bundle ACLR technique was used. Graft choice was according to the surgeon’s preferences. For primary reconstructions performed with HT autograft, the semitendinosus tendon was harvested and prepared as a quadrupled graft. If the length or diameter of the graft was considered insufficient (< 8 mm), the gracilis tendon was also harvested. The BPTB autograft was routinely harvested as the central third of the patellar tendon with two bone blocks. Both grafts were routinely fixed using an Endobutton fixation device (Smith & Nephew, Andover, Mass, USA) on the femoral side and Ethibond no. 2 sutures (Ethicon Inc, Somerville, New Jersey, USA) tied over an AO bicortical screw (Smith & Nephew, Andover, Mass, USA) with a washer as a post or using an interference screw on the tibial side. All the patients followed a standardized rehabilitation protocol. The early rehabilitation phase focused on reducing the swelling, regaining range of motion and gait correction. For all patients, quadriceps strengthening was restricted to closed kinetic chain exercises during the first 3 months. Based on muscle strength, coordination and functional performance, the patients were allowed to return to sports 6 months postoperatively at the earliest.

### Laxity

Anterior knee laxity was assessed preoperatively and at the 6-month follow-up by experienced physiotherapists, at our outpatient clinic, using the KT-1000 arthrometer (MEDmetric, Corp., San Diego, CA, USA) [44]. A 134-N anterior tibial load, at 20° of knee flexion, was applied. At least three measurements for each knee were performed and the median value was registered. The anterior tibial translation (ATT) reduction from preoperative to postoperative for the ACL-reconstructed knee and the preoperative and postoperative difference in displacement (side-to-side, STS, difference) between the ACL-injured knee and the healthy knee were expressed in millimeters. The postoperative STS difference values were then stratified into three different groups according to the International Knee Documentation Committee knee examination form [22] and “surgical failure” was defined as a STS difference > 5 mm (IKDC grades C and D).

### Patient-reported outcome measures (PROMs)

The Lysholm score [42] was collected preoperatively and at the 6-month follow-up. The Knee injury and Osteoarthritis Outcome Score (KOOS) [33] was collected preoperatively and 1 year postoperatively.

### Correlation laxity—PROMs

The correlation between postoperative knee laxity and PROMs, for both grafts, was studied by directly comparing the postoperative KOOS and Lysholm score between the three groups with a STS difference ≤ 2 mm, between 3 and 5 mm, and > 5 mm.

Ethical permission for this study was obtained by the regional ethics committee, Karolinska Institutet (Diarienumber 2016/1613-31/32).

### Statistical analysis

A statistician performed all the data analyses using IBM SPSS Statistics (Version 23.0, IBM Corp, Armonk, New York, USA). Demographic variables were summarized with standard descriptive statistics, such as frequency, mean (M) and standard deviation (SD). Differences between the groups at baseline were analyzed with Student’s *t* test for continuous variables and Pearson’s *χ*^2^-test for categorical variables. Differences between the graft groups in terms of the mean preoperative and postoperative laxity values, laxity changes (described as ATT reduction) and PROMs were analyzed with an analysis of variance (ANOVA) for repeated measurements, with age, gender, meniscal injuries and cartilage injuries (only for PROMs) as covariates, since there were significant differences in these variables between the graft groups at baseline. The postoperative STS difference between the injured and non-injured knee was dichotomized into two classes, normal (≤ 5 mm) and failures (> 5 mm), and differences in distribution between the two graft groups were analyzed with Pearson’s *χ*^2^-test for categorical variables. The level of significance was 5% (two-tailed) for all analyses.

## Results

A cohort of 5462 patients, composed of 692 BPTB and 4770 HT autografts, with complete preoperative and postoperative instrumented laxity measurements, was eligible for the analysis. The mean (SD) time from the knee injury to the surgical procedure was 15 (11.4) months.

Demographic data for both graft groups are presented in Table 1.

**Table 1:** Demographic data at the index operation (*N* = 5462 patients)

	BPTB (*n* = 692)	HT (*n* = 4770)	*P* value
Age, year, mean ± SD	28.8 ± 8.4	28.1 ± 10.5	0.03
Male	498 (71.9)	2552 (53.5)	< 0.001
Concomitant injuries			
Meniscus	192 (27.8)	1932 (40.5)	< 0.001
Cartilage	80 (11.6)	893 (18.7)	< 0.001

Data are reported as *n* (%), unless otherwise indicated

*BPTB* bone–patellar tendon–bone, *HT* hamstring tendon, *SD* standard deviation

### Laxity

**Fig. 1:**
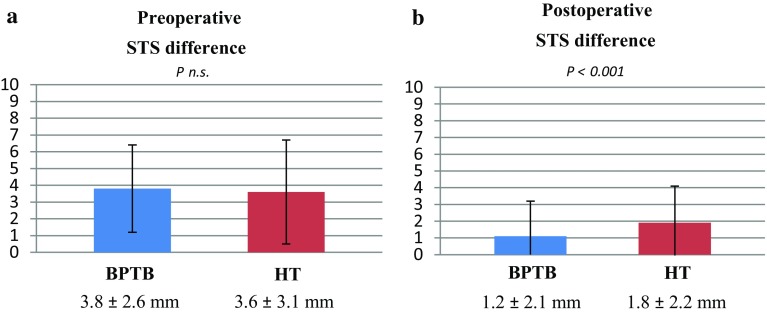
Mean ± SD preoperative and postoperative side-to-side KT-1000 arthrometer measurements. A significantly greater laxity was found for the HT graft postoperatively. Covariates applied to the model are age, gender and meniscal injuries. *STS* side-to-side, *BPTB* bone–patellar-tendon–bone, *HT* hamstring tendons

Preoperatively, the mean anterior STS difference between the injured knee and the healthy knee was 3.8 mm (SD 2.6) and 3.6 mm (SD 3.1) for BPTB and HT graft, respectively (n.s.). At 6 months postoperatively, the mean anterior STS difference was significantly reduced to 1.2 mm (SD 2.1) (*P* < 0.001) for the BPTB graft and to 1.8 mm (SD 2.2) (*P* < 0.001) for the HT graft. However, the HT group showed a significantly increased postoperative knee laxity compared with the BPTB group (Fig. 1a, b).

**Fig. 2:**
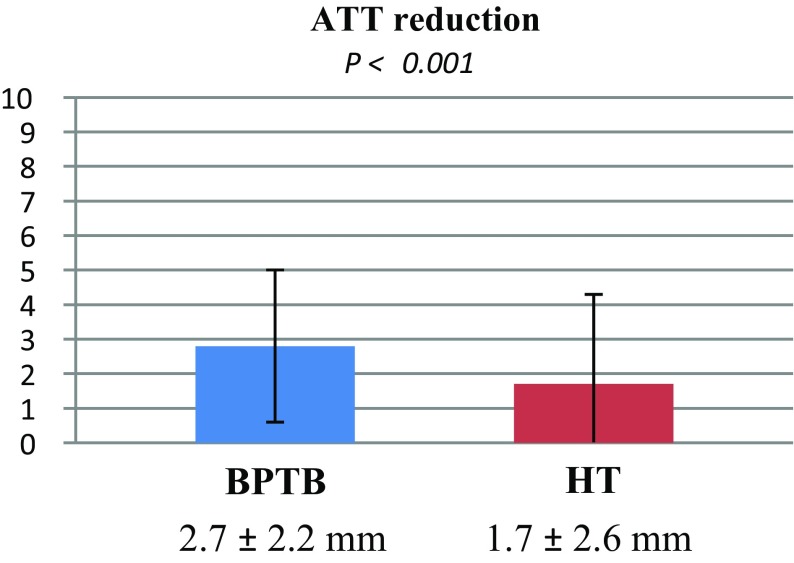
Mean ± SD ATT reduction from preoperatively to postoperatively measured with the KT-1000 arthrometer. A significantly greater reduction was found for the BPTB graft. Covariates applied to the model are age, gender and meniscal injuries. *ATT* anterior tibial translation, *BPTB* bone–patellar tendon–bone, *HT* hamstring tendons

The mean ATT reduction from preoperative to postoperative, in the ACL-reconstructed knee, was significantly larger for the BPTB graft compared with the HT graft (Fig. 2).

Postoperative KT-1000 STS values were stratified according to the International Knee Documentation Committee knee examination form [22]. Significant differences were found between the graft groups, with more surgical failures for the HT autograft (Table 2).

**Table 2:** Stratified KT-1000 arthrometer side-to-side difference values in both graft groups at follow-up

Graft	No.	Patients, no. (%)		
		≤ 2 mm	3–5 mm	> 5 mm (Surgical failures)
BPTB	692	519 (75.0%)	156 (22.6%)	17 (2.4%)
HT	4770	3037 (63.7%)	1527 (32.0%)	206 (4.3%)^a^

^a^A significantly higher rate of surgical failures (> 5 mm) was found in the HT group (P < 0.001) vs the BPTB group

### Patient-reported outcome measures (PROMs)

The mean improvement in the Lysholm score from preoperatively to the 6-month follow-up was significant for both graft groups (*P* < 0.001), but no significant differences in improvement were found between the grafts.

The mean improvement from preoperatively to the 1-year follow-up in all KOOS subscales was analyzed for both grafts. All subscales improved significantly in both graft groups (*P* < 0.001), but four of five subscales revealed significant differences between the grafts. The amelioration was significantly greater for Pain, Activities of Daily Living, Quality of Life and, in particular, the Sports subscale for the HT graft compared with BPTB graft. No significant differences between the grafts were found for the Symptoms subscale (Table 3).

**Table 3:** Improvement in the Knee Injury and Osteoarthritis Outcome Score (KOOS) and Lysholm Score

	BPTB				HT				Mean improvement (BPTB vs HT)^b^ *P* value
	No.	Preoperative	Follow-up^a^	Mean improvement	No.	Preoperative	Follow-up^a^	Mean improvement	
KOOS									
Pain	625	79.0 ± 14.9	87.0 ± 11.8	8.0	4741	79.8 ± 15.5	89.3 ± 11.5	9.5	0.02
Symptoms	625	75.6 ± 16.9	82.2 ± 14.9	6.6	4740	75.7 ± 17.2	82.2 ± 25.6	6.5	n.s.
ADL	615	88.0 ± 13.0	93.7 ± 8.8	5.7	4734	88.2 ± 14.0	95.4 ± 8.4	7.2	0.006
Sports	593	48.8 ± 24.7	64.1 ± 21.8	15.3	4507	50.7 ± 26.6	74.9 ± 21.8	24.2	< 0.001
QOL	606	35.9 ± 17.5	58.0 ± 19.7	22.1	4608	38.3 ± 21.4	64.1 ± 22.1	25.8	0.001
Lysholm Score	660	70.1 ± 15.7	84.6 ± 12.1	14.5	4005	70.0 ± 15.9	84.0 ± 12.6	14.0	n.s.

Data are presented as the mean ± SD

*No*. number of patients with scores available preoperatively and postoperatively, *BPTB* bone–patellar tendon–bone, *HT* hamstring tendons, *ADL* Activities of Daily Living, *QOL* Quality of Life

^a^Six months for the Lysholm score, 1 year for the KOOS

^b^Covariates applied to the model are age, gender, meniscal and cartilage injuries

### Correlation laxity—PROMs

**Fig. 3:**
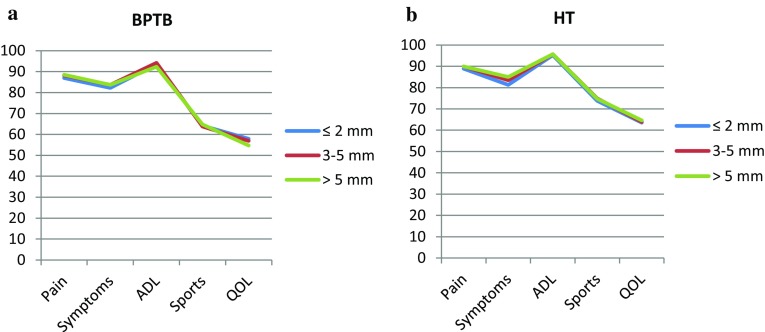
Knee injury and Osteoarthritis Outcome Score (KOOS) in each stratified side-to-side laxity group, for both grafts, at the 1-year follow-up. *BPTB* bone–patellar tendon–bone, *HT* hamstring tendons, *ADL* Activities of Daily Living, *QOL* Quality of Life

**Fig. 4:**
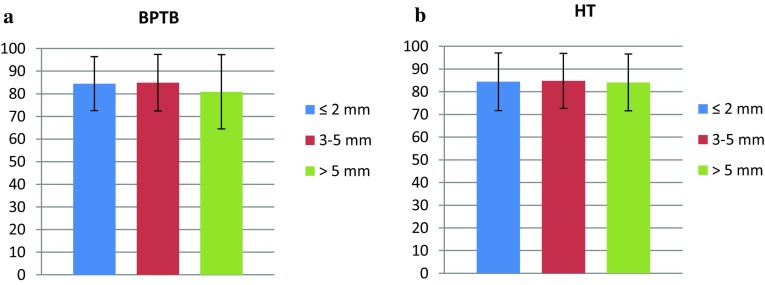
Lysholm score in each stratified side-to-side laxity group, for both grafts, at the 6-month follow-up. *BPTB* bone–patellar tendon–bone, *HT* hamstring tendons

No differences in the KOOS or Lysholm score were found between the stratified STS laxity groups for either BPTB or HT autograft (Figs. 3a, b, 4a, b).

## Discussion

The principal finding of the present study was that ACLR performed with HT autograft results in greater postoperative anterior knee laxity and more surgical failures (STS > 5 mm) compared with BPTB autograft. The BPTB autograft showed a significantly larger reduction in anterior knee laxity (ATT reduction) with primary ACLR. These results are in accordance with some previous research reporting more stable knees after ACLR using BPTB graft [3, 8, 11, 29].

Both grafts showed a significant improvement in all the studied PROMs from preoperatively to the postoperative follow-up. No differences in improvement regarding the Lysholm score were found between the two grafts. Conversely, significant differences in improvement were found for the KOOS Pain, Activities of Daily Living, Quality of Life and, in particular, the Sports subscale in favor of the HT graft, despite the larger postoperative knee laxity for this graft group. No correlation between the postoperative anterior knee laxity, KOOS or Lysholm score was found for both graft groups.

The main goals of ACLR are to restore knee laxity and improve subjective knee function. Abnormal knee laxity is a risk factor for subjective instability, meniscal injuries and the early onset of osteoarthritis [36]. Several prospective studies have compared the postoperative anterior knee laxity after ACLR, using BPTB and HT autografts, and have reported contradictory results [1, 3, 11, 23, 43]. However, many of these studies comprise a relatively small number of patients and may not have sufficient power to detect differences between the grafts. In their meta-analysis, Goldblatt et al. [18] reported data on 182 patients (91 HT, 91 BPTB) for KT-1000 manual maximum side-to-side difference, concluding that the use of BPTB was associated with a more stable knee. Li et al. [29] analyzed a total of 518 patients (276 HT, 242 BPTB) included in six trials in which the postoperative KT-1000 arthrometer at 89 N was used. They observed a statistical difference in favor of BPTB autografts. Conversely, in a more recent meta-analysis comprising 858 patients (422 BPTB, 436 HT) with KT1000/2000 values available, Xie et al. [45] suggested that the HT autograft produces similar results compared with the BPTB autograft in terms of knee laxity. Our study, which comprised a significantly larger number of patients treated at a single center with a standardized surgical procedure, rehabilitation protocol and laxity assessment, shows that the use of HT autograft is associated with a significantly increased anterior knee laxity compared with BPTB autograft after ACLR.

The laxity of the knee following ACLR is probably influenced by the biomechanical and histologic properties of the graft. As ACL surgery developed in the 1980s and 1990s, the BPTB graft was considered as the “gold standard”, due to its stiffness and high failure load found in studies at that time [6, 31]. Enthusiasm for the HT graft developed when later studies showed its low donor-site morbidity and that a four-strand HT graft is stronger and stiffer than a 10-mm BPTB graft [19, 20]. This in vitro evidence suggested that the hamstring tendons could have better mechanical properties. However, the main factor affecting the structural strength of the graft during the first postoperative period is not the initial graft strength itself. The fixation points on the femoral side and even more on the tibial side appear to be the weakest points [39]. The historical advantage of the BPTB graft is that it offers rigid fixation and rapid osteo-integration inside the femoral and tibial tunnels with its patellar and tibial bone plugs, whereas the HT graft has always presented some concerns regarding the tendon-to-bone healing process and lack of rigid fixation [12]. The increased postoperative knee laxity could, therefore, be a clinical manifestation of the different biomechanical properties and the slower ligamentization process of the HT graft [30]. In addition, it has been shown that knee laxity after ACLR does not change with both grafts over time [17, 34, 38]. Feller et al. [9] compared BPTB and ST autografts in 57 patients at 4 and 8 months and at 1- to 3-year follow-ups. The side-to-side difference was measured using the KT-1000 arthrometer at 67 and 134 N. No decrease or increase in laxity was seen at any follow-up.

Even if a difference in improvement in four of five KOOS subscales was found in favor of HT graft, these differences are small and probably not clinically significant, except perhaps for the Sports subscale. The minimum clinically important difference (MCID) for the KOOS is considered to be 8–10 points for all subscales [26]. The highest difference in improvement between the two grafts was found in the Sports subscale, with an improvement of 24.2 for the HT graft compared with 15.3 for the BPTB graft. These differences between the grafts could be explained by the “donor-site morbidity” associated with BPTB autograft. Other authors have reported greater anterior knee pain and kneeling pain after ACLR performed with BPTB compared with HT at a short-term follow-up [29, 45], that, however, disappear with time [35].

It was hypothesized that patients with greater postoperative knee laxity would experience poorer subjective knee outcomes. Instead, our findings showed no association between postoperative anterior knee laxity and patient-reported outcome measures (KOOS and Lysholm score) for either BPTB or HT graft.

Although we showed that postoperative anterior knee laxity does not correlate with subjective knee function in the short-term, the greater laxity in the ACL-reconstructed knee could have negative effects. Struewer et al. [41] found a significant correlation between a higher degree of osteoarthritis and increased anterior knee laxity measured with the KT-1000 arthrometer. Greater risks of graft failure and ACL revision [11, 14] but also a higher incidence of additional knee surgery [15, 37] have been described with the use of HT graft compared with BPTB graft for primary ACLR. An explanation could be that the increased laxity with HT graft makes the knee more vulnerable to new traumas or could be responsible for greater “stress” on the graft itself that is inclined to failure. At the same time, it is also possible that patients with HT autograft who, in the present study, showed a significantly greater improvement in the Sports KOOS subscale compared with those with BPTB autograft experience a more satisfactory subjective knee function and are, therefore, more prone to take part in activities with a risk of ACL re-ruptures and new injuries.

In recent years, in Scandinavia, the graft choice for ACLR has shifted towards favoring HT autograft rather than BPTB autograft [14]. In Denmark, the use of HT autograft increased from 68% of all graft types in 2005 to 85% of all graft types in 2011 [32]. In 2012, 95% of the primary ACLRs were performed using HT autografts in Sweden [27]. The reason for the increased popularity of the HT autograft, despite the proposed higher risk of re-rupture compared with the BPTB autograft [14], could be its potential inferior postoperative morbidity [18, 29, 45].

There are advantages and disadvantages for each graft. Graft choice should be individualized according to several factors, such as the graft potential for restoring knee laxity, patient demands, and graft morbidity.

The principal strength of the present study is the analysis of a large cohort (5462 patients). Moreover, all patients received surgery, rehabilitation and preoperative and postoperative assessments at the same institution. This makes this study different from previous studies based on national registries or meta-analyses.

Several limitations are present. First, even if a standardized surgical technique has been used at our institution, the study timeframe is long and some surgical variables could have changed over time. Although the vast majority of cases were performed with the anteromedial drilling technique, it was not possible to perform a thorough analysis of the surgical technique used. However, it has been shown that no relevant differences exist in terms of subjective knee function and anteroposterior laxity between the transtibial and anteromedial drilling techniques [21, 40]. Second, the indications for the choice of graft are unknown, with the inevitable consequence of possible selection bias. Third, there is a lack of information regarding rotational laxity, which has been associated with outcomes [24].

The findings of the present study provide clinicians with valuable information regarding differences in knee laxity and subjective knee function between BPTB and HT autograft after primary ACLR. The use of BPTB autograft should be considered for patients with high knee stability demands.

## Conclusions

Primary ACLR performed with HT autograft resulted in greater postoperative anterior knee laxity and significantly more surgical failures (STS > 5 mm) compared with BPTB autograft. The BPTB autograft resulted in a greater anterior knee laxity reduction (ATT reduction) with primary ACLR. The HT autograft led to a significantly greater improvement in the KOOS Pain, Activities of Daily Living, Sports and Quality of Life subscales compared with BPTB autograft. However, apart from the Sports subscale, these differences are too small to be considered clinically relevant. There was no correlation between postoperative anterior knee laxity and patient-reported outcome measures for both grafts.
